# A Systematic Review on the Effects of Different Types of Probiotics in Animal Alzheimer's Disease Studies

**DOI:** 10.3389/fpsyt.2022.879491

**Published:** 2022-04-27

**Authors:** Tanja J. de Rijke, M. H. Edwina Doting, Saskia van Hemert, Peter P. De Deyn, Barbara C. van Munster, Hermie J. M. Harmsen, Iris E. C. Sommer

**Affiliations:** ^1^Department of Biomedical Sciences of Cells and Systems, University Medical Center Groningen/University of Groningen, Groningen, Netherlands; ^2^Department of Medical Microbiology and Infection Prevention, University Medical Center Groningen/University of Groningen, Groningen, Netherlands; ^3^Winclove Probiotics, Amsterdam, Netherlands; ^4^Alzheimer Center Groningen, University Medical Center Groningen, Groningen, Netherlands; ^5^Department of Neurology, University Medical Center Groningen, Groningen, Netherlands; ^6^Department of Internal Medicine, University Medical Center Groningen, Groningen, Netherlands

**Keywords:** microbiota-gut-brain axis, gut microbiota composition, short chain fatty acids, inflammatory markers, amyloid-beta, cognitive functioning, *Bifidobacterium longum*, *Clostridium butyricum*

## Abstract

Alzheimer's disease (AD) is a global public health priority as with aging populations, its prevalence is expected to rise even further in the future. The brain and gut are in close communication through immunological, nervous and hormonal routes, and therefore, probiotics are examined as an option to influence AD hallmarks, such as plaques, tangles, and low grade inflammation. This study aimed to provide an overview of the available animal evidence on the effect of different probiotics on gut microbiota composition, short chain fatty acids (SCFAs), inflammatory markers, Amyloid-β (Aβ), and cognitive functioning in AD animal models. A systematic literature search was performed in PubMed, SCOPUS, and APA PsychInfo. Articles were included up to May 2021. Inclusion criteria included a controlled animal study on probiotic supplementation and at least one of the abovementioned outcome variables. Of the eighteen studies, most were conducted in AD male mice models (*n* = 9). Probiotics of the genera *Lactobacillus* and *Bifidobacterium* were used most frequently. Probiotic administration increased species richness and/or bacterial richness in the gut microbiota, increased SCFAs levels, reduced inflammatory markers, and improved cognitive functioning in AD models in multiple studies. The effect of probiotic administration on Aβ remains ambiguous. *B. longum* (NK46), *C. butyricum*, and the mixture SLAB51 are the most promising probiotics, as positive improvements were found on almost all outcomes. The results of this animal review underline the potential of probiotic therapy as a treatment option in AD.

## Introduction

Alzheimer's disease (AD) is the most common form of dementia and worldwide an estimated 44 million people suffer from AD (1). The global prevalence is expected to rise in the future due to aging populations. AD is therefore a global public health priority (2). Key symptoms of AD include cognitive decline, behavioral changes, and inability to perform ordinary tasks. AD is associated with decreased quality of life and limited daily functioning (3, 4). The cause of AD is still unknown, although insights into the molecular pathology do exist. One of the pathological hallmarks of AD is the formation of plaques in the brain, consisting of the peptide amyloid-beta (Aβ) (5). Aβ is also produced in a healthy brain. In the case of AD, however, the production or decreased removal of Aβ results in the formation of plaques which negatively affects the communication between brain cells (5). Also, oligomeric Aβ negatively affects the function and structure of synapses (6). Synaptic markers are found to predict cognitive functioning in AD (7). Another pathological hallmark of AD is the formation of neurofibrillary tangles in the brain, consisting of highly phosphorylated tau protein (8). These fibrillary inclusions are reported to be accountable for neuronal cell death (8).

The microbiome consists of a diverse ecosystem of microorganisms, including bacteria, fungi, protozoa, viruses, and all their genes and functions, in contrast to the microbiota which only comprises living microbes (9). The amount and diversity of the microbiota gradually decrease with age (10, 11). At all ages, variability and heterogeneity of the gut microbiota differs largely between persons due to extrinsic factors (e.g., diet, antibiotics, lifestyle, or disease) and intrinsic factors (e.g., genetics) (9, 10, 12). In general, high species diversity is interpreted as a sign of a healthy microbiome. The human microbiome has been linked to multiple aspects of human health and disease, including AD (13–15). There is a bidirectional relationship between the brain and the gut, which is also known as the “gut-brain axis” (GBA) (16). The GBA is bidirectional in the sense that the gut microbiota signals to the brain and the brain signals to the gut microbiota (17). The brain and gut communicate via neural processing of the central nervous system and the enteric nervous system (17). The GBA connects the emotional and cognitive parts of the brain with peripheral intestinal functions and mechanisms, such as immune activation, intestinal permeability, enteric reflex, and entero-endocrine signaling (17). One way by which the gut microbiota exerts its effects is through the production of metabolites, such as short chain fatty acids (SCFAs) and branched-chain amino acids, and bacterial fragments, such as peptidoglycans, that reach the brain via the circulation (10). Therefore, the GBA is sometimes also referred to as the microbiota-gut-brain axis (9, 18).

Recent studies have focused on the role of the gut microbiota in several brain disorders, including autism, anxiety, schizophrenia, Parkinson's disease, and AD (10). When focusing on AD, emerging evidence hypothesizes that gut dysbiosis is suggested to stimulate the aggregation of Aβ, neuroinflammation, oxidative stress, and insulin resistance (19). However, a causal relation between gut dysbiosis and neural dysfunction remains elusive until now (19). On a group level, the gut microbiome of people with AD was different compared to healthy age- and sex-matched individuals (14, 20, 21). For instance, Vogt et al. (14) found that the gut microbiome of people with AD was less diverse. Several studies comparing AD patients to healthy controls reported a reduction in the gut microbiome in the phylum *Firmicutes* and in the genus *Bifidobacterium (B.);* and an increase in *Bacteroidetes* and *Proteobacteria*, more specifically the phylum *Enterobacteriaceae* (14, 20). Additionally, a correlation was found for genera that were more abundant in AD compared to controls, with CSF markers of AD pathology (14). Liu et al. (20) also found a significant correlation between clinical severity scores of people with AD and altered microbiomes. For instance, a negative association was found between Mini-Mental State Examination (MMSE) and Montreal Cognitive Assessment (MoCA) scores and the phylum Proteobacteria, its class Gammaproteobacteria, and the family *Enterobacteriaceae* (*P* < 0.05) and between MoCA scores and *Veillonellaceae* (*P* < 0.05) (20). In contrast, a positive association was found between Clinical Dementia Rating and the family *Enterobacteriaceae* (*P* < 0.05), and between MMSE scores and Bacteroidetes and *Ruminococcaceae* (*P* < 0.05) (20). In addition, the intestinal barrier and the blood-brain barrier (BBB) may both play an important role in the pathogenesis of AD. The permeability of both barriers increases with age (22). This increased permeability, as well as damage to the intestinal barrier and BBB caused by gut dysbiosis, may facilitate the entry of pathogens into the blood and brain (23). These pathogens can theoretically enter the brain through the damaged BBB and can worsen neuroinflammation and induce amyloid aggregation, which is a primitive immune reaction (23). Consequently, increased intestinal permeability and microbial dysbiosis also trigger systemic inflammation within the body, for example, by increasing serum interleukin 6 (IL-6) levels (23). Elevated levels of IL-6 are also found in serum and brain tissue of people with AD (24). Systemic inflammation in AD is also argued to induce proinflammatory states of microglia and astrocytic phenotypes, which stimulate tau hyperphosphorylation, Aβ oligomerization, component activation, and the breakdown of neurotransmitters into potentially toxic metabolites (25).

Probiotics are products that deliver live microorganisms with a suitable viable count of well-defined strains with a reasonable expectation of delivering benefits for the host's wellbeing (26). Health benefits have been demonstrated for several probiotic strains, including *Lactobacillus* (*L*.), and *Bifidobacterium* (*B*.) (27). It should be noted that the efficacy of probiotics is strain- and disease-specific (28), hence many types of bacteria could be considered probiotics under the right conditions. The benefits of probiotics occur in the GI tract by influencing the intestinal microbiota and the introduction of beneficial functions to the microbiota, which could result in the prevention or amelioration of gut inflammation or other systemic disease phenotypes (29). Besides the positive influences on the human gut microbiota, probiotics can regulate neurotransmitters and growth factors, such as gamma-aminobutyric acid, serotonin, glutamate, and brain-derived neurotrophic factor (30, 31). Moreover, the gut microbiota is not only important for the intestinal permeability, but also for the production of SCFAs (32). SCFAs that are produced through probiotics are suggested to induce a decrease in pro-inflammatory cytokines, due to their immunomodulatory effects (32, 33). For example, SCFAs provide anti-inflammatory effects in the intestinal mucosa through the inhibition of histone deacetylases and the activation of cell surface G-protein coupled receptors in intestinal epithelial cells and immune cells (34). Two recent meta-analyses investigated the effect of probiotic supplementation in patients with AD or mild cognitive impairment. One study reported improvement in cognitive functioning with the use of probiotics, and it was hypothesized that this was due to the decrease in levels of inflammatory and oxidative biomarkers (35). The other study looked at the effectiveness of probiotic supplementation on cognitive functioning in people with dementia and found no beneficial effect of probiotic supplementation on cognitive functioning in patients with AD, with very low evidence certainty (36). Both analyses included only three RCTs on AD, of which one used co-supplementation with selenium, which precludes any firm conclusions on the potential benefits of probiotics for AD.

Several findings have emerged from animal studies that used different models of AD, which may provide information that could be translated to a clinical application. A systematic review on animal evidence can provide an overview, which may help to find an optimal (mix of) probiotic(s) to further test in AD patients in clinical trials. Moreover, more insights into the potential underlying mechanism can be provided by investigating the effect of probiotics on multiple outcome variables related to AD and/or the microbiota-gut-brain-axis. This systematic review aims to provide an overview of the available animal evidence on the effect of different strains of probiotics on gut microbiota composition, SCFAs, inflammatory markers, Aβ, and cognitive functioning in models of AD.

## Methods

### Search Strategy

**Figure 1:**
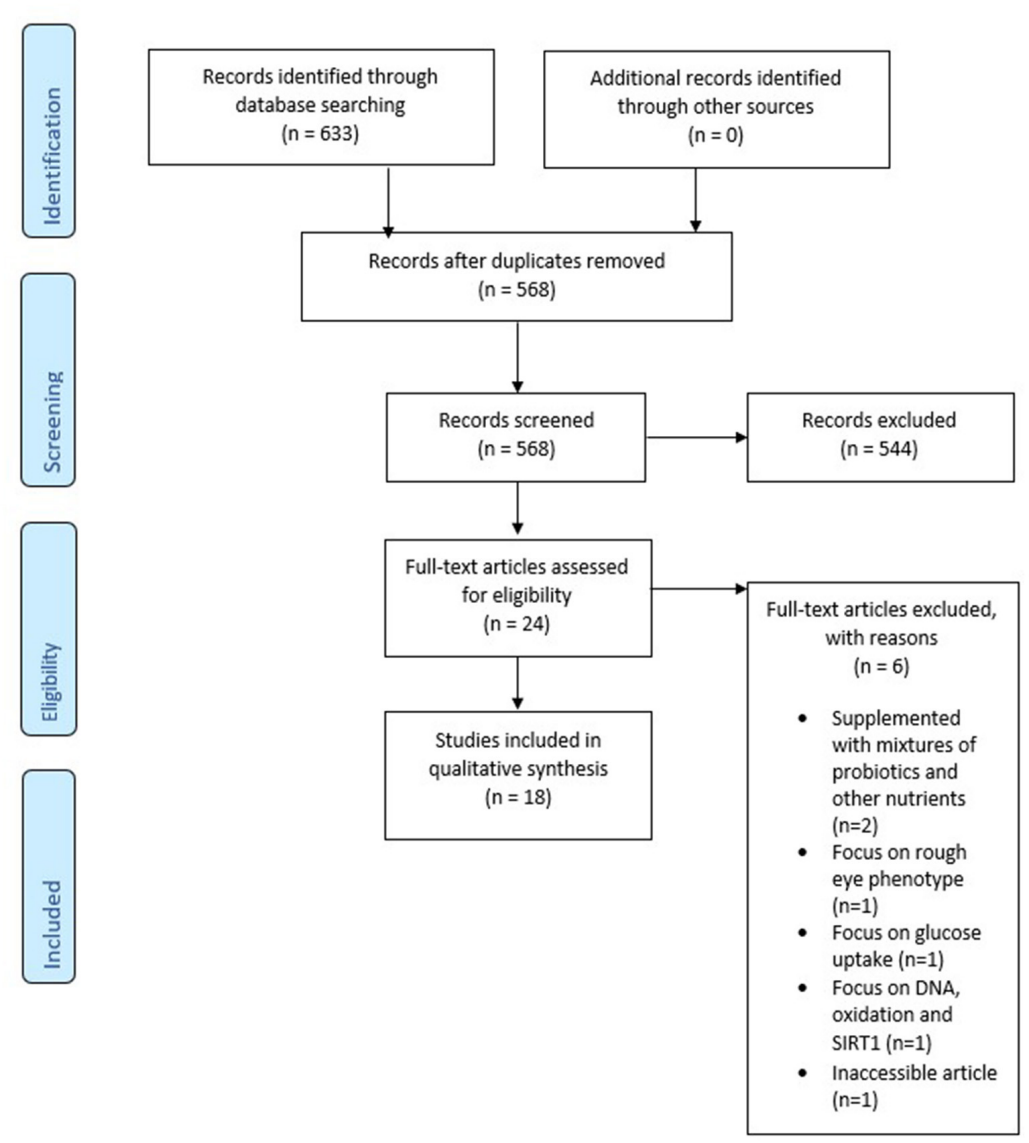
PRISMA flow chart.

This study was performed according to the Preferred Reporting for Systematic Reviews and Meta-analysis (PRISMA) (37). A systematic literature search was performed in PubMed, SCOPUS, and APA PsycInfo. Articles were included up to May 2021. Combinations of the following keywords were used: “probiotics” and “Alzheimer's disease.” These words were added to the search query together with synonyms or MeSH terms. Duplicates have been removed from the final study selection. After selecting relevant titles and abstracts selection, full-text articles were assessed. A PRISMA flow chart is used to graphically display the final selection of articles (see Figure 1).

### Inclusion and Exclusion Criteria

Full-text articles were considered eligible if they met the following criteria: (1) the article is peer-reviewed, (2) the article is written in English, (3) a controlled study was conducted on probiotic supplementation, (4) the study uses an animal model of AD, and (5) at least one of the following outcome measures was addressed: cognitive functioning, gut microbiota composition, Aβ deposition, inflammatory markers, and SCFAs. Besides gut microbiota composition, gut permeability or other gut health markers were not included due to a lack of available data. The exclusion criterium was when a study uses mixtures of probiotics with other nutrients or interventions, so that the effect of probiotics cannot be distinguished.

### Outcome Measures

The outcome measures of this study included gut microbiota composition, SCFAs, inflammatory markers, Aβ, and cognitive functioning. Gut microbiota composition encompassed species richness, microbial abundance, and microbiome diversity. Inflammatory markers comprised biomarkers that reflect the pro- or anti-inflammatory status, assessed either in blood or CSF. Finally, cognitive functioning was defined as mental abilities, including learning, thinking, reasoning, remembering, problem solving, decision making, and attention (38).

### Data Extraction

Data extraction was done on the following items: publication year, country, study sample characteristics of the intervention and control group (e.g., animal model; control group; sample size; sex), intervention characteristics (e.g., type, duration, and dosage of probiotics; type and duration and dosage of control condition), the operationalization of the outcome measures, and results. The extracted data were compared and analyzed for similarities and differences.

### Quality Assessment

The risk of bias was assessed with SYRCLE (39), because this tool was specifically designed to assess the methodological quality of animal intervention studies. SYRCLE is based on the widely used Cochrane Collaboration Risk of Bias tool and was adapted to aspects of bias that specifically play a role in animal research (39).

Ten entries of potential biases in the included studies were reviewed, which are related to selection bias, performance bias, detection bias, attrition bias, reporting bias and other biases. Each potential bias was evaluated by answering SYRCLE's signaling questions with “yes” indicating a low risk of bias, “no” indicating a high risk of bias, and “no information” indicating an unclear risk of bias (39). A summary score was calculated based on the number of yeses compared to the total number of SYRCLE items.

## Results

### Search Results

The systematic literature search resulted in 633 studies from which 65 duplicates were removed. The title and abstract of 568 articles were screened for in- and exclusion criteria. In total, 24 articles were read in full-text. Out of these, six articles were excluded (40–45), because they were inaccessible, animals were supplemented with mixtures of probiotics with other nutrients, or had a different focus (glucose uptake, rough eye phenotypes, DNA, oxidation and SIRT1), leaving eighteen articles for analysis. The final PRISMA flow chart can be found in Figure 1.

### Quality Assessment

In total, 18 articles were assessed for methodological quality by means of the SYRCLE tool (see Table 1). Although the SYRCLE score does not have an overall quality score, most articles scored a four, five, or six out of ten, which reflects a moderate quality. Two remarkable results can be distinguished: Ou et al. (46) had a relatively high methodological quality with a score of eight out of ten, whereas Lee et al. (62) had a relatively low methodological quality with a score of three out of ten. Most studies had incomplete descriptions of the methodology, resulting in an unclear risk of bias. The high frequency of an unclear risk of bias was especially true for items that measure selection bias, performance bias, detection bias, and attrition bias.

**Table 1:** Methodological quality of included studies based on SYRCLE.

**SYRCLE risk of bias tool**	**1**	**2**	**3**	**4**	**5**	**6**	**7**	**8**	**9**	**10**	**Total score**
Ou et al. (46)	+	+	+	?	+	+	+	?	+	+	8
Kaur et al. (47)	+	+	?	+	?	+	?	?	+	+	6
Wang et al. (48)	+	+	?	+	?	+	?	?	+	+	6
Cao et al. (49)	+	-	?	+	?	+	?	?	+	+	5
Kaur et al. (50)	+	+	?	?	?	+	?	?	+	+	5
Wang et al. (51)	+	+	?	?	?	+	?	?	+	+	5
Sun et al. (52)	+	+	?	?	?	+	?	?	+	+	5
Athari Nik Azm et al. (53)	+	+	?	?	?	+	?	?	+	+	5
Shamsipour et al. (54)	+	+	?	?	?	+	?	?	+	+	5
Guilherme et al. (55)	?	+	?	+	?	+	?	?	+	+	5
Rezaei Asl et al. (32)	-	+	?	?	?	+	?	?	+	+	4
Kobayashi et al. (56)	?	+	?	?	?	+	?	?	+	+	4
Lee et al. (57)	-	+	?	?	?	+	?	?	+	+	4
Cecarini et al. (58)	?	+	?	?	?	+	?	?	+	+	4
Nimgampalle and Kuna (59)	?	+	?	?	?	+	?	?	+	+	4
Wu et al. (60)	+	-	?	?	?	+	?	?	+	+	4
Bonfili et al. (61)	?	+	?	?	?	+	?	?	+	+	4
Lee et al. (62)	?	?	?	?	?	+	?	?	+	+	3

*Item 1, sequence generation (selection bias); item 2, baseline characteristics (selection bias); item 3, allocation concealment (selection bias); item 4, random housing (performance bias); item 5, blinding (performance bias); item 6, random outcome assessment (detection bias); item 7, blinding (detection bias); item 8, incomplete outcome data (attrition bias); item 9, selective outcome reporting (reporting bias); item 10, other sources of bias. + reflects “yes,” - reflects “no,” ? reflects “unclear”*.

### Study Characteristics

An overview of the study characteristics can be found in Table 2. All articles were published between 2017 and 2021. Most studies were conducted in mice models of AD (*n* = 14; mostly APP/PS1), and four studies used AD models in Wistar rats. Only two studies used female animals, compared to thirteen studies using male animals and three studies did not provide a description on the sex of the animals.

**Table 2:** Study characteristics of included preclinical studies on probiotics and AD.

**Reference and country**	**Animal model**	**Probiotics**	**Intervention characteristics**	**Variables**
Kaur et al. (50); USA	Mice: 6-8 m.o. ♀ AppNL-G-F (*n* = 30) & C57BL/6 WT (*n* = 30). Groups: (1) WT, (2) WT+Pro, (3) AD, and (4) AD+Pro.	VSL#3	8 weeks; 0.32 x 10^9^ CFU bacteria/25 g mice; oral adm.	Antibodies and reagents; fecal sample collection; intestinal permeability; gastric emptying and intestinal transit; Aβ and cytokines; western blot analysis; eicosanoid analysis; bile acid analysis; immunohistochemistry; behavior test
Rezaei Asl et al. (32); Iran	R: ♂ Wistar. Groups: (1) control (*n* = 10), (2) AD (*n* = 8), (3) AD + pro (*n* = 8), (4) Sham group (*n* = 10), (5) Pro+control (*n* = 9)	*L. acidophilus; B. bifidum; B. longum*	8 weeks 500 mg; 15 x 10^9^ CFU; intragastric adm.	Behavioral experiments; electrophysiological experiments; fecal bacteria quantification; measurement of biomarkers; plasma concentration of malondialdehyde; brain tissue and histological examination
Cao et al. (49); China	M: 4 m.o. APP/PS1. Groups: (1) AD (*n* = 3), (2) AD+Pro (*n* = 4) & (3) 6 m.o. B6C3F1 wt	*B. Lactis* Probio-M8	45 days (exp.1) and 4 months (exp.2); 1 x 10^9^ CFU/ml at a dose of 0.2 ml/10gr body weight; instragastric adm.	Immunohistochemistry; microbiome profiling; processing of sequencing data; behavioral test
Wang et al. (48); China	M: 6 m.o. ♂ APP/PS1 mice & wt. Groups: (1) control WT (*n* = 15), (2) AD APP/PS1 (*n* = 15), (3) Mem + APP/PS1(*n* = 15), (4) AD + Pro APP/PS1 (*n* = 15), (5) AD + Pro+Mem (*n* = 15)	*L. plantarum* (ATCC 8014)	12 weeks, daily; 1 x 10^9^ CFU/ml; oral adm.	Behavioral experiments; histochemical and biochemical analyses; metagenomic analyses
Kobayashi et al. (56); Japan	M: 10 w.o. ♂ ddY mice	*B. breve* A1	1 x 10^9^ CFU in 0.2 ml; oral adm.	Behavioral tests; physiological analyses; RNA sequencing analysis; microbiota analysis; SCFA analysis
Wang et al. (51); China	M: 8 w.o. ♂ APP/PS1 and wt. Groups: (1) AD group (*n =* 10), (2)AD + Pro(BB) group (*n =* 10), (3)AD + Pro(L.P.) group (*n =* 10), (4)AD + Pro(BB+LP) group (*n =* 10), 5) wt (*n =* 10)	*B. bifidum* (TMC3115) and *L. plantarum* 45 (LP45)	22 weeks; 1 x 10^9^ CFU; oral adm.	Behavioral tests and microbiota analysis
Lee et al. (57); South Korea	M: 4 m.o. ♂ 5XFAD mice and 18 m.o. male C57BL/6 mice. Groups: (1) AD + Pro(*n =* 6), (2) AD (*n =* 6), (3) WT + Pro(*n =* 6), (4) wt (*n =* 6)	*B. longum* (NK46)	6x per week for 8 weeks (AD groups) and 4 weeks (control groups); 1 x 10^9^ CFU/mouse/day; oral adm.	Biochemical parameters; immunostaining, immunoblotting and ELISA; memory behavioral tasks; immunofluorescence assay; immunoblotting; myeloperoxidase activity assay; determination of LPS; culture of fecal bacteria; pyrosequencing
Sun et al. (52); China	M: 6 m.o. APP/PS1 vs. C57BL/6 wt. Groups: (1) Ad group (*n =* 10), (2) AD + Pro (*n =* 10), (3) control	*C. butyricum* (WZMC1016)	Daily for 4 weeks; 1 x 10^9^ CFU ml-1; intragastric adm.	Behavioral evaluation; histology analysis; ELISA assay; butyrate assay; Aβ oligomer preparation; BV2 microglia culture and treatment; immunofluorescence; western blot analysis
Lee et al. (62); South Korea	M: 6 m.o. ♂ 5XFAD	*L. plantarum* (C29) vs. C29-fermented defatted soybean powder	Daily for 2 months; C29: 1 x 10^9^ CFU per mouse and FDS: 200 mg per mouse; oral adm.	Memory behavioral tasks; histological and biochemical parameters.
Cecarini et al. (58); Italy/USA/Brazil	M: 8 m.o. ♂ 3xTg-AD. Groups: (1) untreated mice (T0, *n =* 8), (2) mice treated with lyophilized milk (C, *n =* 8), (3) mice treated with control lyophilized LAB (pExu:empty, *n =* 8), and (4) mice treated with lyophilized p62-LAB (LAB(pExu:p62), *n =* 8).	*L. lactis* subsp. *cremoris* (MG1363) strain (LAB) versus p62-pExu transformed cells (p62-LAB)	Daily for 2 months; 1 x 10^9^ CFU; oral adm.	Novel object recognition; brain tissue; Aβ levels; immunohistochemistry; western blot analysis; oxyblot analysis; proteasome activity assays; cathepsin B and L activities; ghrelin, leptin and GIP, GLP-1; 16SrRNA gene sequencing
Kaur et al. (47); USA	M: 6-8 m.o. ♀AppNL-G-F (*n =* 15) and C57BL/6 wt (*n =* 15). Groups: (1) wild-type vehicle (WT veh), (2) wild-type VSL#3 (WT VSL), (3) AppNL-G-F vehicle (AppNL-G-F), and (4) AppNL-G-F VSL#3 (AppNL-G-FVSL#3)	VSL#3	8 weeks; 0.32 x 10^9^ CFU bacteria/25 gr mice; oral adm.	Microbiome analysis; SCFA analysis; Ki-67 stereology and counting in hippocampus; immunohistochemistry; Aβ; behavioral analysis
Ou et al. (46); China	M: 9 m.o. ♂APP/PS1 and wt mice. APP/PS1 groups: (1) AD + normal chow diet (NCD), (2) AD + NCD + Pro, (3) AD + High Fat Diet (HFD), (4) AD + HFD + Pro) (*n =* 10 per group). WT groups: (1) wt, (2) WT+Pro (*n =* 6 per group)	*A. muciniphila*	Daily for 6 months; 5 x 10 CFU in 200 microliter sterile PBS; oral adm.	MRI; immunohistochemistry and histology; biochemical assays and ELISA; glucose tolerance test; open-field and Y-maze tests; western blot analysis; real-time PCR
Nimgampalle and Kuna (59); India	R: 3 m.o. ♂Wistar. Groups: (1) control (*n =* 6), (2) AD group (*n =* 6), (3) AD+Pro (*n =* 6), (4) Pro (*n =* 6)	*L. plantarum* (MTCC1325)	60 days; 12 x 10^8^ CFU/ml; 10 ml/kg body weight; oral adm.	Morphological features; cognitive behavior; gross behavioral activity; brain tissue; histopathological examination; biochemical estimation of cholinergic system
Wu et al. (60); China	M: Wt and APP/PS1. Groups: (1) WT group, (2) WT+Pro group, (3) AD group, and (4) AD+Pro group	*B. longum* (1714)	Daily for 6 months; 1 x 10^9^ CFU/ml at 0.2 ml/10 g of body mass; oral adm.	Immunohistochemistry; immunofluorescence; Thioflavin S staining; western blot analysis; PCR; Aβ42
Athari Nik Azm et al. (53); Iran	R: 8 w.o. ♂ Wistar. Groups: (1) Control (*n =* 12), (2) control + pro (*n =* 12), (3) sham operation (*n =* 12), (4) AD (*n =* 12), (5) AD + Pro (*n =* 12)	2 grams probiotics mix: *L. acidophilus* (1688FL431-16LA02), *L. fermentum* (ME3), *B. lactis* (1195SL609-16BS01), and *B. longum* (1152SL593)	8 weeks; 500 mg of each with 1 x 10^10^ CFU; oral adm.	Behavioral test; amyloid plaque detection; SOD, CAT activities and MDA level detection in hippocampus tissue; Detection of bacteria count in stool samples
Bonfili et al. (61); Italy	M: 8 w.o. ♂ 3xTg-AD and coetanus wt. Groups: (1) AD (*n =* 32), (2) AD + Pro (*n =* 32), (3) wt (*n =* 32), (4) wt + Pro (*n =* 32)	SLAB51	4 months; 200 bn bacteria/kg/day; oral adm.	Behavioral assessment; microbiota analysis; ELISA assay for ghrelin, leptin and GIP, cytokine analyses, Aβ levels, GLP-1; Congo red staining for Aβ and FGF9 immunohistochemical detection; TUNEL analysis; proteasome activity assays; cathepsin B and L; western blotting analysis
Shamsipour et al. (54); Iran	R: ♂ Wistar (*n =* 40). Groups: (1) control, (2) AD model receiving Aβ, (3) AD rats with MIIT (AD + MIIT), (4) AD + Pro (AD + PROB), and AD receiving bout treatment (AD + MIIT + Pro)	*L. plantarum* and *B. bifidum*	Daily for 8 weeks; 1 x 10^9^ CFU of each strain; oral adm.	Behavioral testing; neuronal cell population assay and molecular studies; ChAT protein assay
Guilherme et al. (55); Germany	M: 4 w.o. ♂5xFAD. Groups: (1) control (*n =* 8), (2) antibiotics group (*n =* 7), (3) probiotics group (*n =* 7)	OptiBac	14 weeks; 1 x 10^9^ CFU/ml; oral adm.	Nest building test; brain tissue analysis; immunohistochemistry and densitometric analysis; serum insulin and glucagon; western blotting

*R., rats; M., mice; ♂, male; ♀, female; w.o., weeks old; m.o., months old; CFU, colony forming unit; Mem, Memantine; exp, experiment; L., Lactobacillus; B., Bifdobacterium; C., Clostridium; A., Akkermansia; VSL#3, L. plantarum, L. delbrueckii subsp. Bulgaricus, L. paracasei, L. acidophilus, B. Breve B. longum, B. infantis, and Streptococcus salivarius subsp. thermophilus; SLAB51, Streptococcus thermophilus DSM 32245, B. lactis DSM 32246, B. lactis DSM 32247, L. acidophilus DSM 32241, L. helveticus DSM 32242, L. paracasei DSM 32243, L. plantarum DSM 32244, and L. brevis DSM 27961; Optibac, L. acidophilus and L. rhamnosus; L. lactis subsp. cremoris, Lactobacillus lactis subsp. cremoris; adm., administration; CC, correlation coefficient; ROCC, Receiver Operating Characteristic Curves; AUCV, Area Under the Curves Values; PCA, Principal Component Analysis; Sig, significance*.

Regarding the type of probiotics, lactobacilli and bifidobacteria were frequently used (i.e., *L. plantarum, B. bifidum*, and *B. longum*). Ten studies used a single-strain probiotic, compared to eight studies using a mixture. Two studies used the mixture VSL#3, which consists of *L. plantarum, L. delbrueckii* subsp. *Bulgaricus, L. paracasei, L. acidophilus, B. breve B. longum, B. infantis*, and *Streptococcus salivarius* subsp. *thermophilus*. One study used SLAB51, which is a probiotic mixture consisting of: *Streptococcus thermophilus* (DSM 32245), *B. lactis* (DSM 32246), *B. lactis* (DSM 32247), *L. acidophilus* (DSM 32241), *L. helveticus* (DSM 32242), *L. paracasei* (DSM 32243), *L. plantarum* (DSM 32244), and *L. brevis* (DSM 27961). Another study used the mixture Optibac, which consists of *L. acidophilus* and *L. rhamnosus*. An overview of the other mixtures can be seen in Table 2. Study durations ranged from 4 weeks to 6 months. In fifteen studies, probiotics were administered orally, while in three studies probiotics were administered intragastrically.

### Overall Effects of Probiotics

The majority of studies investigated the effect of probiotics on multiple outcome variables. Two studies looked at all the included outcome variables, whereas one study only looked at one outcome variable (see Table 3). In all studies, probiotics influenced at least one outcome variable of AD. Probiotic administration affected gut microbiota composition (*n* = 13 studies), SCFAs (*n* = 4 studies), inflammatory markers (*n* = 7 studies), and cognitive functioning (*n* = 12 studies). For Aβ, conflicting results were found, as some (*n* = 10 studies) found positive effects, whereas others (*n* = 5 studies) found no effect or no significant effect compared to AD control animals.

**Table 3:** Overview of results per study and probiotic strain on cognitive functioning, gut microbiota composition, Aβ, inflammatory markers, or SCFAs in AD animals compared to AD animals without probiotic administration.

**Significant improvement → // Study and type of probiotic↓**	**Gut microbiota composition**	**SCFAs**	**Inflammatory markers**	**Aβ**	**Cognitive function**
Kaur et al. (50); VSL#3	Yes (2/2)	-	Partially (3/4 yes; 1/4 no)	No (2/2)	No (1/1)
Rezaei Asl et al. (32); *L. acidophilus, B. bifidum, & B. longum*	Yes (1/1)	-	-	No (1/1)	Yes (1/2)
Cao et al. (49); *B. lactis* Probio-M8	Yes (5/7)	-	-	Yes (1/1)	Yes (2/3)
Wang et al. (48); *L. plantarum* (ATCC 8014)	-	-	Yes (2/2)	Yes (1/1)	Yes (1/3)
Kobayashi et al. (56); *B. breve A1*	Yes (1/2)	Yes (1/3)	-	-	Yes (1/2)
Wang et al. (51); *B. bifidum* (TMC3115) *and L. plantarum 45* (LP45)	Yes (2/2)	-	-	-	Yes (1/3)
Lee et al. (57); *B. longum* (NK46)	Yes (4/5)	-	Yes (2/2)	Yes (2/2)	Yes (4/4)
Sun et al. (52); *C. butyricum*	Yes (1/3)	Yes (1/1)	Yes (1/1)	Yes (1/1)	Yes (2/2)
Lee et al. (62); *L. plantarum* (C29)	Yes (2/2)	-	Yes (1/1)	Yes (1/1)	Yes (1/4)
Cecarini et al. (58); *L. lactis* subsp. *cremoris*	Yes (1/4)	-	Yes (2/2)	Yes (2/2)	No (1/1)
Kaur et al. (47); VSL#3	Yes (1/1)	Yes (1/1)	-	No (3/3)	-
Ou et al. (46); *A. municiphila*	-	-	-	Yes (1/2)	Yes (2/2)
Nimgampalle and Kuna (59); *L. plantarum* (MTCC1325)	-	-	-	No (1/1)	Yes (1/1)
Wu et al. (60); *B. longum* (1714)	-	-	Yes (1/1)	Yes (2/2)	-
Athari Nik Azm et al. (53); *L. acidophilus, L. fermentum, B. lactis, & B. longum*	Yes (1/1)	-	-	Yes (1/1)	Yes (1/1)
Bonfili et al. (61); SLAB51	Yes (1/1)	Yes (1/1)	Yes (1/1)	Yes (2/2)	Yes (1/4)
Shamsipour et al. (54); *L. plantarum & B. bifidum*	-	-	-	-	No (1/1)
Guilherme et al. (55); Optibac	Yes (1/2)	-	-	No (1/1)	-

*The numbers behind a yes, no or partially, reflect the amount of tests that has been conducted to measure the outcome variable. For instance, yes (1/3) means that in total three tests have been conducted, of which one showed a significant improvement. SCFA, short chain fatty acids; Aβ, amyloid-beta; L., Lactobacillus; B., Bifdobacterium; C., Clostridium; A., Akkermansia; VSL#3, L. plantarum, L. delbrueckii subsp. Bulgaricus, L. paracasei, L. acidophilus, B. Breve B. longum, B. infantis, and Streptococcus salivarius subsp. thermophilus; SLAB51, Streptococcus thermophiles DSM 32245, B. lactis DSM 32246, B. lactis DSM 32247, L. acidophilus DSM 32241, L. helveticus DSM 32242, L. paracasei DSM 32243, L. plantarum DSM 32244, and L. brevis DSM 27961; Optibac, L. acidophilus and L. rhamnosus; L. lactis subsp. cremoris, Lactobacillus lactis subsp. cremoris*.

### Probiotics and Gut Microbiota Composition

Thirteen studies looked at the effect of probiotics on gut microbiota composition, of which eight looked at microbiome richness and/or diversity (see Tables 3, 4). An improvement in both species richness (51, 57, 62) and bacterial diversity (57, 62) was found compared to control group animals. More specifically, a significant difference in α-diversity was found in four studies (49, 50, 57, 62), which was not replicated by Sun et al. (52) and Cecarini et al. (58). Similarly, two studies (51, 57) found a significant increase in β-diversity compared to AD control animals, whereas three others did not (49, 56, 58).

**Table 4:** Overview of results per study and probiotic strain on gut microbiota composition in AD animals compared to AD animals without probiotic administration.

**Significant improvement → // Study and type of probiotic↓**	**Gut microbiota composition**				
	**Species richness**	**Bacterial diversity**	**β-diversity**	**α-diversity**	**Total count of**
Kaur et al. (50); VSL#3				↑	↑ phylum Verrucomicrobia ↑ phylum Actinobacteria
Rezaei Asl et al. (32); L. *acidophilus*, B. *bifidum*, & B. *longum*					↑ viable counts in feces (CFU/gr)
Cao et al. (49); B. *lactis* Probio-M8			*x*	↑	↑ family Ruminococcaceae ↓ genus Parabacteroides distasonis ↓ genus Streptococcus
Kobayashi et al. (56); B. breve A1			*x*		↑ phylum Actinobacteria ↑ family Bifidobacteriaceae ↓ family Odoribacteriaceae ↓ family Lachnospirceae
Wang et al. (51); B. *bifidum* (TMC3115) and L. *plantarum* 45 (LP45)	↑		↑		↑ genus Parabacteroides ↑ genus Acetatifactor ↑ genus Millionella ↓ genus Desulfovibrio ↓ genus Intestinimonas ↓phylum Bacteroidetes
Lee et al. (57); B. *longum* (NK46)	↑	↑	↑	↑	↑ Bacteroidia ↑ phylum Bacteroidetes ↑ family Prevotellaceae ↓ family Pseudomonadaceae ↓phylum Firmicutes ↓ phylum Proteobacteria ↓ genus Clostridium ↓ family Ruminococcaeceae ↓ family Lachnospiraceae ↓ family Helicobacteriaceae
Sun et al. (52); C. *butyricum*				*x*	↑ genus Alloprevotella ↑ family S24-7 ↓ phylum Deferribacteres ↓ family Helicobacteriaceae ↓ genus Helicobacter
Lee et al. (62); L. *plantarum* (C29)	↑	↑		↑	↑ family Lactobacillaceae
Cecarini et al. (58); L. *lactis subsp. cremoris*			*x*	*x*	↑ family Peptococcaceae ↑ family Ruminococcaceae
Kaur et al. (47); VSL#3					↑↓ genus Bacterioides
Athari Nik Azm et al. (53); L. a*cidophilus*, L. *fermentum*, B. *lactis*, & B. *longum*					↑ genus Bifidobacterium ↑ genus Lactobacillus ↓ coliform
Bonfili et al. (61); SLAB51					↑ genus Bifidobacterium spp. ↓ family Campylobacterales (i.e., Helicobacteriaceae)
Guilherme et al. (55); Optibac					↑ family Lactobacillaceae (after 14 days; not significant after 14 weeks)

*↑, a significant increase was found upon probiotic supplementation compared to control group animals. ↓, a significant decrease was found upon probiotic supplementation compared to control group animals. X, no (significant) effect was found upon probiotic supplementation compared to control group animals. L., Lactobacillus; B., Bifdobacterium; C., Clostridium; VSL#3, L. plantarum, L. delbrueckii subsp. Bulgaricus, L. paracasei, L. acidophilus, B. Breve B. longum, B. infantis, and Streptococcus salivarius subsp. thermophilus; SLAB51, Streptococcus thermophiles DSM 32245, B. lactis DSM 32246, B. lactis DSM 32247, L. acidophilus DSM 32241, L. helveticus DSM 32242, L. paracasei DSM 32243, L. plantarum DSM 32244, and L. brevis DSM 27961; Optibac, L. acidophilus and L. rhamnosus; L. lactis subsp. cremoris, Lactobacillus lactis subsp. cremoris*.

An increase in certain bacterial phyla was observed upon probiotic administration (i.e., *Actinobacteria, Verrucomicrobia, Bacteroidetes*) (50, 56). At the family level, an increase in *Peptococcaceae, Ruminococcaceae, Prevotellaceae*, S24-7, and *Lactobacillaceae* was found compared to control group animals (49, 52, 55–58, 62). An increase in *Acetatifactor, Millionella, Alloprevotella, Parabacteroides, Bifidobacterium, Lactobacillus* and *Bacteroidales* was observed compared to AD controls at the genus level (47, 51–53, 61). In contrast in other studies, a decrease at the phylum level was found for *Firmicutes, Bacteroidetes, Proteobacteria*, and *Deferribacteres* compared to control animals (49, 51, 52, 57). Also, at the family level, a decrease was observed for *Odorbacteraceae, Lachnospiraceae, Helicobacteraceae, Ruminococaceae*, and *Pseudomonadaceae* compared to AD controls (52, 56, 57, 61). A decrease compared to AD control animals was also found for *Parabacteroides, Streptococcus, Desulfovibrio, Bacteroidales, Intestinimonas, Clostridium*, and *Helicobacter* at the genus level (47, 49, 51, 52, 57). At the phylum level, no change in the overall *Firmicutes/Bacteroidetes* ratio was found (50).

When investigating the different probiotics, all included probiotics showed at least one effect on the gut microbiota composition (see Tables 3, 4).

### Probiotics and SCFAs

Only four out of 18 studies addressed the effect of probiotics on SCFA levels, in which an improvement in plasma, serum, fecal, and hippocampal SCFA levels was observed compared to AD control animals (see Tables 3, 5) (47, 52, 56, 61).

**Table 5:** Overview of results per study and probiotic strain on SCFAs in AD animals compared to AD animals without probiotic administration.

**Significant improvement → // Study and type of probiotic↓**	**SCFAs**				
	**Fecal**	**Plasma**	**Serum**	**Hippocampal**	**Type of SCFA**
Kobayashi et al. (56); *B. breve A1*		↑			↑ acetate, but not propionate or butylate
Sun et al. (52); *C. butyricum*	↑				↑ butyrate
Kaur et al. (47); VSL#3			↑	↑	Serum: ↑ acetate, butyrate, isobutyrate, propionate, and lactate Hippocampal: ↑ acetate and lactate, but not butyrate, isobutyrate and propionate
Bonfili et al. (61); SLAB51	↑				↑ acetate, propionate, and butyrate

*↑, a significant increase upon probiotic supplementation compared to control group animals. ↓, a significant decrease was found upon probiotic supplementation compared to control group animals. X, no (significant) effect was found upon probiotic supplementation compared to control group animals. SCFAs, short chain fatty acids; B., Bifdobacterium; C., Clostridium; VSL#3, L. plantarum, L. delbrueckii subsp. Bulgaricus, L. paracasei, L. acidophilus, B. Breve B. longum, B. infantis, and Streptococcus salivarius subsp. thermophilus; SLAB51, Streptococcus thermophiles DSM 32245, B. lactis DSM 32246, B. lactis DSM 32247, L. acidophilus DSM 32241, L. helveticus DSM 32242, L. paracasei DSM 32243, L. plantarum DSM 32244, and L. brevis DSM 27961*.

A study by Kaur et al. (47) reported increased serum and hippocampal SCFAs compared to AD control animals. An increase in plasma acetate levels was found (56), as well as an increase in total fecal butyrate levels compared to AD control animals (52). Furthermore, an increase in fecal levels of acetic, propionic, and butyric acid was observed (61).

When looking at the different strains of probiotics, a significant increase in SCFA levels was observed upon administration with *B. breve* A1, the mixture VSL#3, the mixture SLAB51, and *C. butyricum* (see Tables 3, 5).

### Probiotics and Inflammatory Markers

Eight studies investigated the effect of probiotics on relevant inflammatory markers for AD (50–52, 57, 58, 60–62) (see Tables 3, 6). Seven studies found a reduction in proinflammatory markers compared to control group animals. One study found a partial effect: a significant reduction in levels of proinflammatory cytokines in the ileum was found, as well as a significant improvement in serum eicosanoid levels compared to control group animals (50). In contrast, the same study found no significant effect on proinflammatory cytokines or Lipocalin-2 levels in the brain and no significant effect on protein levels in the ileum compared to AD controls (50).

**Table 6:** Overview of results per study and probiotic strain on inflammatory markers in AD animals compared to AD animals without probiotic administration.

**Significant improvement → // Study and type of probiotic↓**	**Inflammatory markers**			
	**Pro-inflammatory cytokines and/or proteins in the brain**	**Pro-inflammatory cytokines and/or proteins in plasma/serum**	**Pro-inflammatory cytokines and/or proteins in the gut**	**Anti-inflammatory cytokines and/or proteins in the brain**
Kaur et al. (50); VSL#3		↓	x	
Wang et al. (48); *L. plantarum* (ATCC 8014)	↓	↓		
Lee et al. (57); *B. longum* (NK46)		↓	↓	
Sun et al. (52); *C. butyricum*	↓			
Lee et al. (62); *L. plantarum* (C29)		↓	↓	
Cecarini et al. (58); *L. lactis* subsp. *cremoris*	↓			↑
Wu et al. (60); *B. longum* (1714)	↓			
Bonfili et al. (61); SLAB51		↓		

*↑, a significant increase upon probiotic supplementation compared to control group animals. ↓, a significant decrease was found upon probiotic supplementation compared to control group animals. ↓, a significant decrease was found upon probiotic supplementation compared to control group animals. X, no (significant) effect was found upon probiotic supplementation compared to control group animals. L., Lactobacillus; B., Bifdobacterium; C., Clostridium; VSL#3, L. plantarum, L. delbrueckii subsp. Bulgaricus, L. paracasei, L. acidophilus, B. Breve B. longum, B. infantis, and Streptococcus salivarius subsp. thermophilus; SLAB51, Streptococcus thermophiles DSM 32245, B. lactis DSM 32246, B. lactis DSM 32247, L. acidophilus DSM 32241, L. helveticus DSM 32242, L. paracasei DSM 32243, L. plantarum DSM 32244, and L. brevis DSM 27961; L. lactis subsp. cremoris, Lactobacillus lactis subsp. cremoris*.

Several studies found a reduction in proinflammatory cytokines in the brain (hippocampus), and blood (plasma) compared to AD control animals, such as IL-1α, IL-2, IL-6, IL-4, IL-12, IL-17, IL-1β, INF-γ, and TNF-α (51, 52, 57, 58, 60–62). Likewise, a decrease in serum eicosanoids, plasma lipopolysaccharide (LPS) levels, and plasma clusterin concentrations was found (50, 51, 57). Also, an increase in the anti-inflammatory cytokine IL-10 was observed in the brain compared to control group animals (58). Kaur et al. (50) did not find a decrease in proinflammatory proteins or protein levels of proinflammatory cytokines in the brain and ileum compared to AD control animals. When looking more specifically at gut inflammation, a reduction in TNF-α in the colon was reported (62). Cyclo-oxygenase 2 expression in the colon, and nuclear factor kappa-light-chain-enhancer of activated B cells (NF-lb.) activation in the colon and in microglial BV-2 cells were found to be decreased compared to control group animals (57, 62).

Most included probiotics showed at least one significant effect on inflammatory markers (see Tables 3, 6). No significant results were found after administration with the mixture VSL#3. Significant improvements were found upon administration with: *L. plantarum* (ATCC8014), *L. plantarum* (C29), *B. longum* (NK46), *B. longum* (1714), the mixture SLAB51*, L. lactis* subsp. *cremoris*, and *C. butyricum*.

### Probiotics and Aβ

A total of 15 studies assessed the effect of probiotics on Aβ parameters (see Tables 3, 7). A reduction in the amount of Aβ plaques in the brain (46, 49, 51, 53), as well as the size of Aβ plaques in the brain (53), Aβ deposition in the brain (46, 52, 60, 61) and the Aβ expression in the hippocampus (57, 62) was found compared to control group animals. More specifically, a decrease in brain levels of Aβ(1-42) (52, 58, 61) and Aβ(1-40) (58) was observed in four studies compared to control group animals. Contrastingly, three studies did not find any effect in the brain (50) and hippocampus (52, 55), or no significant effect in the brain (32, 59) upon probiotic administration on Aβ. Similarly, another study also found no differences in the brain after probiotic administration compared to control group animals for Aβ(1-40) (61).

**Table 7:** Overview of results per study and probiotic strain on Aβ in AD animals compared to AD animals without probiotic administration.

**Significant improvement → // Study and type of probiotic↓**	**Aβ**					
	**Total amount**	**Size**	**Brain**	**Hippocampus**	**Aβ** **(1-42)**	**Aβ** **(1-40)**
Kaur et al. (50); VSL#3			x			
Rezaei Asl et al. (32); *L. acidophilus, B. bifidum, & B. longum*				x		
Cao et al. (49); *B. lactis* Probio-M8	↓					
Wang et al. (48); *L. plantarum* (ATCC 8014)	↓					
Lee et al. (57); *B. longum* (NK46)				↓		
Sun et al. (52); *C. butyricum*			↓		↓	
Lee et al. (62); *L. plantarum* (C29)				↓		
Cecarini et al. (58); *L. lactis* subsp. *cremoris*					↓	↓
Kaur et al. (47); VSL#3	x				x	x
Ou et al. (46); *A. municiphila*	↓		↓			
Nimgampalle and Kuna (59); *L. plantarum* (MTCC1325)				x		
Wu et al. (60); *B. longum* (1714)			↓			
Athari Nik Azm et al. (53); *L. acidophilus, L. fermentum, B. lactis, & B. longum*	↓	↓				
Bonfili et al. (61); SLAB51			↓		↓	x
Guilherme et al. (55); Optibac				x		

*↑, a significant increase upon probiotic supplementation compared to control group animals. ↓, a significant decrease was found upon probiotic supplementation compared to control group animals. X, no (significant) effect was found upon probiotic supplementation compared to control group animals. Aβ, amyloid-beta; L., Lactobacillus; B., Bifdobacterium; C., Clostridium; A., Akkermansia; VSL#3, L. plantarum, L. delbrueckii subsp. Bulgaricus, L. paracasei, L. acidophilus, B. Breve B. longum, B. infantis, and Streptococcus salivarius subsp. thermophilus; SLAB51, Streptococcus thermophiles DSM 32245, B. lactis DSM 32246, B. lactis DSM 32247, L. acidophilus DSM 32241, L. helveticus DSM 32242, L. paracasei DSM 32243, L. plantarum DSM 32244, and L. brevis DSM 27961; Optibac, L. acidophilus and L. rhamnosus; L. lactis subsp. cremoris, Lactobacillus lactis subsp. cremoris*.

When looking at the different strains of probiotics, no effect regarding Aβ was found upon administration with *L. plantarum* (MTCC1325); the mixture VSL#3; *L. acidophilus, B. bifidum & B. longum*; and Optibac (see Tables 3, 7). Significant effects were observed upon administration with *L. plantarum* (ATCC8014); *L. plantarum* (C29); *B. longum* (NK46); *B. longum* (1714)*; B. lactis* Probio-M8; *Akkermansia municiphila;* the mixture SLAB51*; L. lactis* subsp. *cremoris; C. butyricum*; and *L. acidophilus, L. fermentum, B. lactis* & *B. longum*.

### Probiotics and Cognitive Functioning

Fifteen studies investigated the effect of probiotics on cognitive functioning, of which twelve studies found at least one improvement in cognitive functioning compared to the control groups (see Tables 3, 8) (32, 46, 48–54, 56–59, 61, 62). As shown in Table 3, not all tests performed found an improvement (e.g., only in one out of three tests). Six studies found an improvement for all tests, three of which used more than one test.

**Table 8:** Overview of results per study and probiotic strain on cognitive functioning in AD animals compared to AD animals without probiotic administration.

**Significant improvement → // Study and type of probiotic↓**	**Cognitive function**					
	**Working memory**	**Learning and memory capacity**	**Recognition memory**	**Locomotor activity**	**Anxiety responses**	**Functional integrity of sensory and motor systems**
Kaur et al. (50); VSL#3	x					
Rezaei Asl et al. (32); *L. acidophilus, B. bifidum, & B. longum*	↑					
Cao et al. (49); *B. lactis* Probio-M8	↑		x			x
Wang et al. (48); *L. plantarum* (ATCC 8014)	↑		x	x		
Kobayashi et al. (56); *B. breve A1*		↑		x		
Wang et al. (51); *B. bifidum* (TMC3115) *and L. plantarum 45* (LP45)	↑		x			
Lee et al. (57); *B. longum* (NK46)	↑	↑	↑	↑		
Sun et al. (52); *C. butyricum*	↑		↑			
Lee et al. (62); *L. plantarum* (C29)	↑					
Cecarini et al. (58); *L. lactis* subsp. *cremoris*			x			
Ou et al. (46); *A. municiphila*				↑		
Nimgampalle and Kuna (59); *L. plantarum* (MTCC1325)	↑					
Athari Nik Azm et al. (53); *L. acidophilus, L. fermentum, B. lactis, & B. longum*	↑					
Bonfili et al. (61); SLAB51		x	↑	x	x	
Shamsipour et al. (54); *L. plantarum & B. bifidum*		x				

*↑, a significant increase upon probiotic supplementation compared to control group animals. ↓, a significant decrease was found upon probiotic supplementation compared to control group animals. X, no (significant) effect was found upon probiotic supplementation compared to control group animals. L., Lactobacillus; B., Bifdobacterium; C., Clostridium; A., Akkermansia; VSL#3, L. plantarum, L. delbrueckii subsp. Bulgaricus, L. paracasei, L. acidophilus, B. Breve B. longum, B. infantis, and Streptococcus salivarius subsp. thermophilus; SLAB51, Streptococcus thermophiles DSM 32245, B. lactis DSM 32246, B. lactis DSM 32247, L. acidophilus DSM 32241, L. helveticus DSM 32242, L. paracasei DSM 32243, L. plantarum DSM 32244, and L. brevis DSM 27961; L. lactis subsp. cremoris, Lactobacillus lactis subsp. cremoris*.

Improvements in spatial working memory, as assessed by the Morris Water Maze test, were found after probiotic supplementation compared to control group animals in all studies (32, 48, 51–53, 57, 59, 62). However, a probe trial test, part of the Morris Water Maze test that measures how long the test subject spends in the target quadrant, did not show significant differences between AD rats with probiotic supplementation and control rats (32). Learning and memory capacity, as measured by the Passive Avoidance Test, improved in two studies compared to control group animals (56, 57), whereas it did not improve in another study (61). Inconsistent results were also found for the Novel Object Recognition Test, which measures recognition memory: an improvement was observed in three studies (52, 57, 61), while four other studies did not find any differences compared to AD controls (48, 49, 51, 58). Improvements (46, 57), as well as no difference (56), were found for locomotor activity assessed via the Y-maze test compared to AD control animals. Similarly, the Open-Field test, which measures general locomotor activity, improved compared to AD controls in one study (46), which was not replicated in two other studies (48, 61). No differences compared to AD controls could be assessed for the Elevated Plus Maze Test, which measures anxiety responses (61).

When looking at different strains of probiotics, no effect on cognitive functioning was found upon administration with VSL#3, *L. lactis* subsp. *cremoris*, and *L. plantarum* & *B. bifidum* (see Tables 3, 8). Significant effects were observed upon administration with *B. breve A1*; *L. plantarum* (ATCC8014); *L. plantarum* (C29); *L. plantarum* (MTCC1325); *B. longum* (NK46); *L. acidophilus, B. bifidum* & B. *longum; B. lactis* Probio-M8; *Akkermansia municiphila;* SLAB51*; Clostridium butyricum (C. butyricum)*; and *L. acidophilus, L. fermentum, B. lactis* & *B. longum*.

## Discussion

### Potential Mechanisms to Explain the Effects of Probiotics in AD

AD pathology has been associated with alterations in the gut microbiota composition and GI inflammation (13, 50, 63, 64). It can be argued that increased gut permeability allows increased concentrations of LPS in the gut and in the circulation, which in turn can trigger amyloid secretion in the gut, while amyloid secretion in the gut can exacerbate the intestinal permeability (46, 65, 66). This process results in increased production and translocation of cytokines and pro-inflammatory components of GI origin into the body (46, 65, 66). These inflammatory compounds could not only increase systemic inflammation, but could also cross the BBB and may induce neuroinflammation, amyloid secretion, neuronal injury, dysfunction of specific brain regions, the development of insulin resistance and ultimately lead to neuronal death in AD (46, 65, 67–70). Some animal studies reported a significant decrease in GI inflammation and attenuated intestinal permeability upon probiotic administration in AD model animals (50, 62). Another study argued that probiotic administration could ameliorate cognitive decline by means of a reduction of gut microbiota LPS production and the regulation of microbiota LPS-mediated NF-κB activation in BV-2 cells, a type of microglial cells (57). From a clinical perspective, a recent meta-analysis summarizing human RCTs found a significant improvement in cognition, as well as a significant reduction in high-sensitivity C-reactive protein levels in the probiotics group compared to the control group (35), supporting the idea that probiotics can reduce systemic inflammation. Overall, these findings suggest that probiotic supplementation suppresses the downward spiral of GI inflammation, altered gut microbiota composition, increased gut permeability, translocation of pro-inflammatory compounds through the BBB, and potential adverse inflammatory and metabolic processes in the brain.

Another underlying mechanism might be SCFA production of the metabolites SCFAs by the gut microbiome. Some metabolites, like SCFAs, can pass through the BBB directly, induce a decrease in pro-inflammatory cytokines in the brain, and modulate the maturation of microglia (32, 52, 56, 71). Additionally, SCFAs are found to interfere with protein-protein interactions, which are necessary for the formation of toxic soluble Aβ aggregates/converse Aβ peptides into Aβ neurotoxic aggregates (72). This interference capability is especially true for valeric acid, butyric acid, and propionic acid (72). This way, SCFAs may help to alleviate elements of the pathophysiological processes of AD.

This review found that probiotic administration in AD rodent models increased plasma, fecal, and hippocampal SCFA levels, reduced inflammatory markers in the blood and brain, and improved cognitive functioning in multiple studies, which is consistent with the hypotheses mentioned above.

### *B. longum* (NK46), *C. butyricum*, and the Mixture SLAB51

*B. longum* (NK46), *C. butyricum*, and the mixture SLAB51 seem to be the most promising probiotics for AD, as they have shown the most positive outcomes. *B. longum* is an anaerobic, non-halophilic, gram-positive bacterium that is naturally present in the human GI tract (73). *B. longum* is considered safe by the EFSA (74). Although multiple health benefits have been found upon *B. longum* administration, such as diarrhea prevention in antibiotic treated patients, and immune stimulation (73), it is unclear why this probiotic is effective for AD hallmarks in animal studies. A possible rationale is that *B. longum* (NK46) may stimulate the production of butyrate. Another hypothesis is that *B. longum* is associated with reduced intestinal inflammation and improved epithelial barrier integrity, and therefore decreases the passage of pro-inflammatory compounds through the BBB and reduces other potential adverse metabolic processes (50). *B. longum* is able to decrease gut microbiota LPS production as well as regulate LPS-induced NF-κB activation in microglial BV-2 cells in AD mice (57).

*C. butyricum* is an anaerobic, gram-positive, spore-forming bacteria that is common in the human colon (75). It has various implications for human health, ranging from pathogenic to beneficial, such as inducing botulism in infants and helping to overcome antibiotic-associated diarrhea in children (76, 77). Furthermore, *C. butyricum* is known for its ability to produce large amounts of SCFAs, such as butyrate and acetate (75). It is hypothesized that this high production of SCFAs is the reason for the beneficial health effects of *C. butyricum* in rodent studies. In Asia, *C. butyricum* is frequently used as a probiotic (75). However, *C. butyricum* is not yet on the QPS safety list of the EFSA (74). A recent animal study showed a neuroprotective effect of *C. butyricum* in mouse models of traumatic brain injury, partially due to increased secretion of glucagon-like peptide 1 (GLP-1), a 30-amino acid peptide hormone, through the GBA (78). Stoeva et al. (79) hypothesize that *C. butyricum* stimulates the secretion of GLP-1, which protects the BBB, potentially via the modulation of tight junctions. Likewise, *C. butyricum* is found to prevent brain endothelial barrier dysfunction, as demonstrated by decreased brain water content and the restoration of normal levels of tight junction protein expression (79). Furthermore, significant improvements in neurological dysfunction, brain edema, neurodegeneration, and BBB impairment were observed after *C. butyricum* administration (78). Moreover, *C. butyricum* decreased plasma-d lactate and colonic IL-6, whilst protecting the intestinal barrier integrity and upregulating the expression of occludin (78). Another animal study, which looked at vascular dementia in mice, found that *C. butyricum* significantly ameliorated cognitive dysfunction and histopathological changes, increased brain-derived neurotrophic factor (BDNF) and Bcl-2 levels, which are cell survival proteins that inhibit apoptosis (80), decreased Bax levels, which is part of the Bcl-2 family (81), decreased p-Akt levels, which is phospholyrated protein kinase B, and reduced neuronal apoptosis (79, 82). *C. butyricum* was also found to restore butyrate levels in feces and brain and regulate the gut microbiota in mouse models of vascular dementia (82). When looking at mouse models of AD, administration of *C. butyricum* prevented cognitive impairment, Aβ deposits, microglia activation, and production of TNF-α and IL-1β in the brain of AD mice (52). Also, *C. butyricum* treatment reversed abnormal gut microbiota and butyrate (52). More specifically, butyrate treatment was found to reduce CD11b and Cyclo-oxygenase 2 levels, and suppress the phosphorylation of NF-κB p65 in the Aβ-induced BV2 microglia (52). However, in this research, only one study investigated the effect of *C. butyricum* on AD mice, meaning that no firm conclusions can be drawn upon it yet.

Like mentioned before, SLAB51 is a probiotic mixture consisting of: *Streptococcus thermophilus* (DSM 32245), *B. lactis* (DSM 32246), *B. lactis* (DSM 32247), *L. acidophilus* (DSM 32241), *L. helveticus* (DSM 32242), *L. paracasei* (DSM 32243), *L. plantarum* (DSM 32244), and *L. brevis* (DSM 27961). All probiotics, except *B. lactis*, are considered safe by the EFSA (74). An animal study in AD mice found that the SLAB51 mixture significantly decreased oxidative stress in AD mice brains by activating Sirtuin 1 (SIRT1)-dependent mechanisms (45). SIRT1 is a protein family that is used during the cellular response to inflammatory, metabolic, and oxidative stressors (83). These proteins play a role in NAD^*^ dependent deacetylation of histones, as well as in neuronal plasticity, cognitive function, and neuronal degeneration (84). SIRT1 levels were found to be lower in serum samples of patients with AD or MCI compared to age matched controls (85). Additionally, beneficial antioxidant effects were found in the brain of AD mice after SLAB51 administration (45). Another study in AD mice found that the mixture SLAB51 ameliorated the impaired glucose metabolism in AD by restoring the brain levels of glucose transporters GLUT3 and GLUT1, ameliorating brain glucose homeostasis, reducing tau phosphorylation by modulating pAMPK and pAkt, and decreasing advanced glycation end products (43). Because of this, the authors argue that amelioration of the impaired glucose metabolism in AD is able to delay AD progression through gut microbiota manipulation with SLAB51 (43). From a broader perspective, the SLAB51 mixture is also considered a promising candidate for the prevention or (coadjuvant) treatment of Parkinson's disease, as the mixture was able to protect dopaminergic neurons and improve behavioral impairments in *in-vivo* studies (86). Also, the mixture SLAB51 counteracted neuroinflammation and oxidative stress in both *in-vivo* and *in-vitro* studies (86). Moreover, the mixture SLAB51 modulated the BDNF pathway, increased neuroprotective protein levels, and decreased neuronal death proteins in the *in-vitro* studies (86).

No clinical trials have been found that investigated the effect of *B. longum* (NK46), *C. butyricum*, or the mixture SLAB51 in AD or MCI patients yet. From a broader perspective, two clinical trials investigated a mixture containing bifidobacteria and lactobacilli in AD patients and found significant improvements in cognitive functioning and some metabolic parameters (87, 88). One of these clinical trials used co-supplementation with selenium. In contrast, a recent meta-analysis, that included these two studies and an RCT in patients with severe AD (89), found no effect on cognitive function in AD patients upon probiotic supplementation, which all consisted of lactobacilli and bifidobacteria (36).

### Combined Interventions

Although in this review we looked exclusively to interventions with only probiotics, in the future it may be wise to look broader than probiotics alone, since the effects on AD hallmarks appear to be stronger when probiotic supplementation is combined with another intervention. This insight may imply that probiotics work synergistically with other interventions. For instance, a stronger effect was found upon probiotic administration together with memantine (1 mg/ml), which is an AD drug classified as an N-Methyl-D-aspartic acid receptor antagonist, exercise, *L. plantarum*-fermented soybean, and p62-transformed *L. lactis* compared to probiotics alone (51, 54, 58, 62). Taking this chain of thought further, this review focused exclusively on probiotics due to the lack of animal studies on prebiotics. Prebiotics are substrates (often carbohydrates) that are selectively utilized by host microorganisms that confer a health benefit. There are multiple types of prebiotics that are suggested to benefit the microbiota (90–92). Prebiotics metabolized by the gut microbiota to SCFAs are, like probiotics, suggested to slow down AD progression due to their effect on the intestinal microbiota (33, 72). One animal AD study, investigating the prebiotic effects of fructooligosaccharides, found that these specific fructooligosaccharides could, among other things, improve oxidative stress and inflammation, regulate the synthesis and secretion of neurotransmitters, positively affect the diversity and stability of the microbiome of AD rats, and down-regulate the expression of both tau and Aβ1-42 (93). From a broader perspective, healthy dietary patterns characterized by high levels of prebiotics and probiotics, in association with other nutrients, are found to delay cognitive decline and decrease the risk of AD (66, 94).

### Translation to Clinical Randomized Controlled Trials

This review is conducted from an animal perspective. Therefore, results from this study cannot be one-on-one translated into the clinic. This is not only due to the dissimilarity of AD animal models compared to AD patients, but also due to confounders in animal experiments, such as environmental factors and host genetic background (95). This review is also based on studies that used a variety of AD models, which could have introduced some bias by potentially affecting the composition of the gut microbiota differently (95). In addition, both the human microbiome (96) and AD characteristics (97) are sex-specific, and thus, women and men may benefit differently from probiotic augmentation. Most animal studies used male rodents and separate studies are needed to investigate females.

In this review, four studies used AD rat models (32, 53, 54, 59). Three of these studies used a mixture of bifidobacteria and lactobacilli strains, while one study used *L. plantarum*. Interestingly, the findings are relatively in line with the results of mice studies in the sense that significant evidence can be found for improvement on both cognitive functioning and gut microbiota composition, whereas more ambiguous results can be found for Aβ levels.

To go from animal to clinical trials, information on preliminary efficacy, toxicity, safety, and pharmacokinetics is needed. Research should look into the optimal duration of probiotic supplementation in AD, as well as the ability of probiotics to survive passage through the human GI tract. For future clinical trials, it is recommended to use oral administration (i.e., in the form of a sachet or pill), as this is minimally invasive for patients.

### Limitations, Strengths and Recommendations for Future Research

Some limitations of this review must be mentioned. First, the included studies used a variety of animal models, as well as a variety of cognitive tests. These cognitive tests assess a slightly different part of cognitive functioning, which negatively influences the internal validity of this review. Second, as assessed in Chapter 3.2, only one study (46) has a high methodological quality. Therefore, the general poor methodological quality may have negatively influenced the results of this study. These findings support the statement that the reporting of experimental details on animals, methods and materials in animal studies is often poor (98). Third, the included studies used relatively small intervention groups and mostly male mice, which both negatively affect the external validity of the results. This is unfortunate as sex and gender differences exist both in the human form of AD and in the human microbiome. Furthermore, most of the included studies were conducted over a period of 2 months. Therefore, long-term conclusions cannot be drawn. Also, specific probiotics were only tested in a single study, and no direct comparisons between probiotics have been made so far. Lastly, publication bias may have caused an underreporting of studies where no effects were found. A strength of this review is that this review provides an in-depth overview of all animal studies on AD on probiotic strain level, whilst taking multiple outcome variables into account.

Future research should investigate the clinical effects of probiotic supplementation on AD symptoms and hallmarks. *B. longum* (NK46), *C. butyricum* and the mixture SLAB51 are promising types of probiotics to test in clinical trials. *B. longum* is considered safe for human consumption by the EFSA (74). It is advised to use oral administration. It is also argued that AD drugs can potentially have a negative effect on the gut microbiota whilst temporarily alleviating the symptoms of AD (e.g., cognitive improvement, reduced inflammation, or a reduction of Aβ and tau proteins) (94). Others argue that the combined administration of prebiotics, probiotics, and treatment could prevent or alleviate gut problems, which may potentially strengthen the efficacy of AD drugs by eliminating a possible factor that sustains the disease (94). This combination may be especially relevant to investigate in AD patients who are currently unresponsive to pharmacological treatment (94).

## Conclusion

In conclusion, this review shows that probiotic administration in AD rodent models increased species richness and/or bacterial diversity of the gut microbiota, increased SCFA levels, reduced inflammatory markers, and improved cognitive functioning in multiple studies. The effect of probiotic administration on Aβ remains ambiguous. *B. longum* (NK46), *C. butyricum*, and the mixture SLAB51 are the most promising probiotics, as positive improvements were found on almost all outcomes. *B. longum* is considered safe for human consumption by the EFSA. A drawback is that each of these probiotics was tested in only one study. It would be helpful when the findings can be repeated by other groups and/or in other models. Taking all studies into account, this animal review underscores the potential of probiotic therapy as a treatment option in AD and this topic warrants animal and clinical follow-up.

## Data Availability Statement

The original contributions presented in the study are included in the article/supplementary material, further inquiries can be directed to the corresponding author/s.

## Author Contributions

TR designed the study and performed the literature search, screening, data extraction, data analysis, and manuscript writing. SH, PD, BM, HH, MD, and IS contributed to the manuscript writing. All authors have approved the final version of this manuscript and contributed to the process of making arguments within the manuscript.

## Funding

This research was part of the project No Guts No Glory. This project was supported *via* an anniversary grant of the Dutch Brain Foundation (Hersenstichting) Grant No. 94648.

## Conflict of Interest

SH was employee of Winclove Probiotics. Winclove produces, markets and investigates probiotics. HH and Winclove receive collective research grants. The remaining authors declare that the research was conducted in the absence of any commercial or financial relationships that could be construed as a potential conflict of interest.

## Publisher's Note

All claims expressed in this article are solely those of the authors and do not necessarily represent those of their affiliated organizations, or those of the publisher, the editors and the reviewers. Any product that may be evaluated in this article, or claim that may be made by its manufacturer, is not guaranteed or endorsed by the publisher.

## Abbreviations

Aβ: amyloid beta
AD: Alzheimer's disease
BBB: blood-brain barrier
Bcl-2: B-cell lymphoma 2
BDNF: brain-derived neurotrophic factor
ELISA: enzyme-linked immunosorbent assay
GBA: gut-brain axis
GI: gastrointestinal
IL: interleukin
INF-γ: interferon gamma
LPS: lipopolysaccharides
MoCa: Montreal Cognitive Assessment
MMSE: Mini-Mental State Examination
NF-κB: nuclear factor kappa-light-chain-enhancer of activated B cells
SCFA: short-chain fatty acid
TNF-α: tumor necrosis factor alpha.
